# Beraprost ameliorates postmenopausal osteoporosis by regulating Nedd4-induced Runx2 ubiquitination

**DOI:** 10.1038/s41419-021-03784-8

**Published:** 2021-05-15

**Authors:** Huo-Liang Zheng, Wen-Ning Xu, Wen-Sheng Zhou, Run-Ze Yang, Peng-Bo Chen, Tao Liu, Lei-Sheng Jiang, Sheng-Dan Jiang

**Affiliations:** Department of Clinic of Spine Center, Xinhua Hospital, Shanghai Jiaotong University School of Medicine, 200082 Shanghai, China

**Keywords:** Proteolysis, RNAi

## Abstract

Bone health requires adequate bone mass, which is maintained by a critical balance between bone resorption and formation. In our study, we identified beraprost as a pivotal regulator of bone formation and resorption. The administration of beraprost promoted differentiation of mouse bone mesenchymal stem cells (M-BMSCs) through the PI3K–AKT pathway. In co-culture, osteoblasts stimulated with beraprost inhibited osteoclastogenesis in a rankl-dependent manner. Bone mass of p53 knockout mice remained stable, regardless of the administration of beraprost, indicating that p53 plays a vital role in the bone mass regulation by beraprost. Mechanistic in vitro studies showed that p53 binds to the promoter region of neuronal precursor cell-expressed developmentally downregulated 4 (Nedd4) to promote its transcription. As a ubiquitinating enzyme, Nedd4 binds to runt-related transcription factor 2 (Runx2), which results in its ubiquitination and subsequent degradation. These data indicate that the p53–Nedd4–Runx2 axis is an effective regulator of bone formation and highlight the potential of beraprost as a therapeutic drug for postmenopausal osteoporosis.

## Introduction

Osteoporosis is an age-related bone disease that is associated with an increased risk of fractures^1^. Fractures caused by osteoporosis impose a significant burden on the society and the economy^2^. Postmenopausal osteoporosis accounts for a large proportion of patients with osteoporosis^3^. Osteoporosis is mainly caused by an imbalance in osteoblast-mediated bone formation and osteoclast-mediated bone resorption^4^. Osteoclasts originate from hematopoietic precursor cells and are mainly responsible for absorbing the formed bone, whereas osteoblasts originate from mesenchymal stem cells (MSCs) and are involved in the formation of new bone^5,6^. In postmenopausal patients with osteoporosis, the rate of bone resorption exceeds that of bone formation, resulting in decreased bone mass^7^.

The drugs currently used for osteoporosis are mainly of two types: drugs that promote bone formation, such as human parathyroid hormone^8,9^, and drugs that inhibit bone resorption, such as bisphosphonates (alendronate, zoledronate) and receptor activator of nuclear factor kappa-B ligand (rankl) neutralizing antibody (denosumab)^8,10–12^. Romosozumab is a new type of anti-osteoporosis drug that increases bone mass and reduces fracture risk^13,14^. Although substantial progress has been made in the treatment of osteoporosis worldwide, it is particularly important to further study the physiological and pharmacological mechanisms that control bone remodeling.

Beraprost is used clinically for the treatment of arterial occlusion^15,16^. Misoprostol, a similar prostaglandin receptor agonist, has positive effects in treatment of osteoporosis^17^. Identifying new uses of existing drugs such as beraprost is valuable for further development and understanding of drug effects. Beraprost increased the bone mass of ovariectomized mice, which may be due to the enhancement of osteogenic function and inhibition of osteoclast function. The administration of beraprost also increased the concentration of serum P1NP and 1,25-hydroxyvitamin in postmenopausal women^18^. Sequencing results showed that the PI3K–AKT signaling pathway may play a critical role in the regulation by beraprost. PI3K/AKT pathway is involved in bone development^19–21^. P53 is the most sensitive gene to beraprost’s stimulation and plays an important role in bone metabolism^22,23^. Down-regulation of p53 significantly promoted the differentiation of osteoblasts and inhibited the differentiation of osteoclasts through the RANKL signaling axis. However, the effect of beraprost in increasing bone mass was weakened and almost absent in p53 knockout mice. Thus, our study identified p53 as an effective regulator of beraprost-mediated bone remodeling. The gene expression of Nedd4 correlated with the expression of p53. Mdm2–p53–Nedd4-2 signaling plays a critical role in the regulation of neural network synchrony^24^. We found that p53-regulated Nedd4 expression and induced its transcription in M-BMSCs. Here, we further explored the function of Nedd4 in osteoblasts using co-IP assays. We found that M-BMSCs overexpressing Nedd4 exhibited a weakened bone mass phenotype and Nedd4 negatively regulated the expression of Runx2 by promoting the ubiquitination of Runx2. In conclusion, our data demonstrate that beraprost is a potentially novel target for the treatment of osteoporosis.

## Results

### Beraprost treatment prevents bone loss induced by ovariectomy (OVX)

To study the role of beraprost in estrogen deficiency-induced osteoporosis, we first developed sham and OVX models using young female C57 mice (10-week-old). Two weeks after the operation, the ovariectomized mice were administered beraprost (0.1 µg/30 g) or vehicle (saline) daily by intragastric route for 5 weeks.

**Fig. 1:**
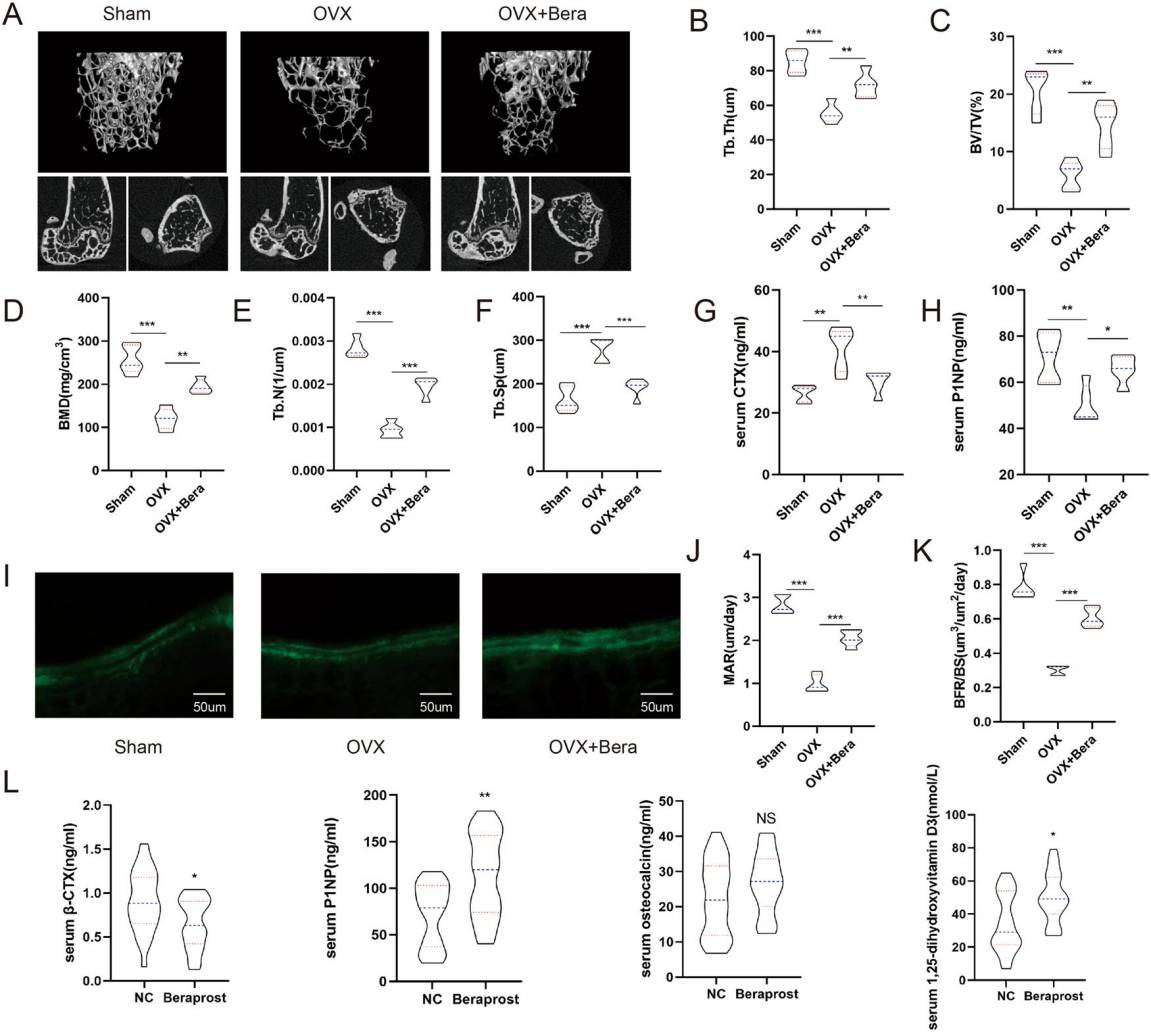
Beraprost treatment prevents bone loss induced by ovariectomy. **A** Representative images of micro-CT reconstruction of distal femurs. **B**–**F** Quantitative analyses of micro-CT of distal femurs. Results are shown as mean ± standard deviation (SD); *n* = 5; *P < 0.05, **P < 0.01, and ***P < 0.001 by analysis of variance (ANOVA) with Tukey’s post-hoc test. BMD bone mineral density (mg/cm^3^); Tb.N trabecular number (1/µm), Tb.Sp trabecular separation (µm), Tb.Th trabecular thickness (µm), BV/TV bone volume (%). **G**, **H** Serum concentration of β-CTX and P1NP. Results are shown as mean ± SD; *n* = 5; **P < 0.01, ***P < 0.001 by ANOVA with Tukey’s post-hoc test. **I** Representative images of calcein double-labeled distal femurs. Scale bars = 50 μm. **J**, **K** Quantification of mineral apposition rate (MAR, µm/day), and bone-formation rate over bone surface (BFR/BS, µm^3^/µm^2^/day). Results are shown as mean ± SD; *n* = 5; ***P < 0.001 by ANOVA with Tukey’s post-hoc test. **L** Concentrations of β-CTX, P1NP, osteocalcin, and 1,25-dihydroxvitamin in serum of postmenopausal women. Results are shown as mean ± SD; *n* = 26, *P < 0.05, **P < 0.01 by *t* test.

To determine whether the surgery was successful, the uterine weights were measured. The uterine weights were reduced by nearly 90% in the OVX groups compared to that in the sham groups, indicating that the ovary was successfully removed. Micro-CT analysis of the distal femur metaphysis suggested that the trabecular bone volume per tissue volume (BV/TV), bone mineral density (BMD), trabecular thickness (Tb.Th), and trabecular number (Tb.N) in OVX groups was significantly decreased compared to that in the sham group, and these indexes returned to higher levels in mice treated with beraprost (Fig. 1A–F). Enzyme-linked immunosorbent assay (ELISA) was used to detect cytokine markers of bone formation and resorption in the serum. The bone resorption marker, β crosslinked C-telopeptide of type 1 collagen (β-CTX), was lower in beraprost-treated mice, whereas the serum levels of the bone formation marker procollagen type 1 N-terminal propeptide (P1NP) was increased (Fig. 1G, H). The dynamic bone formation rate evaluated by calcein double-labeling in mice administered beraprost was higher than that in the OVX group (Fig. 1I–K).

### Beraprost promotes bone metabolism in postmenopausal patients

Patients were divided into two groups. Each group consisted of 26 women who were menopausal for more than 5 years. One group was treated with beraprost, while the other group did not receive beraprost and was considered the control group. All patients were evaluated for age, body mass index (BMI), duration of menopause, and other risk factors (such as taking hormones or anti-osteoporosis drugs within the past year, and other diseases that affect bone quality). The bone metabolism index of each patient was recorded. Interestingly, we found that serum osteocalcin (OCN) did not change significantly between the two groups. However, the concentration of serum P1NP and 1,25-hydroxyvitamin increased slightly while the concentration of serum β-CTX decreased in the beraprost-treated group, indicating the potential of beraprost in the treatment of osteoporosis (Fig. 1L).

### Beraprost promotes the regeneration of bone defects

**Fig. 2:**
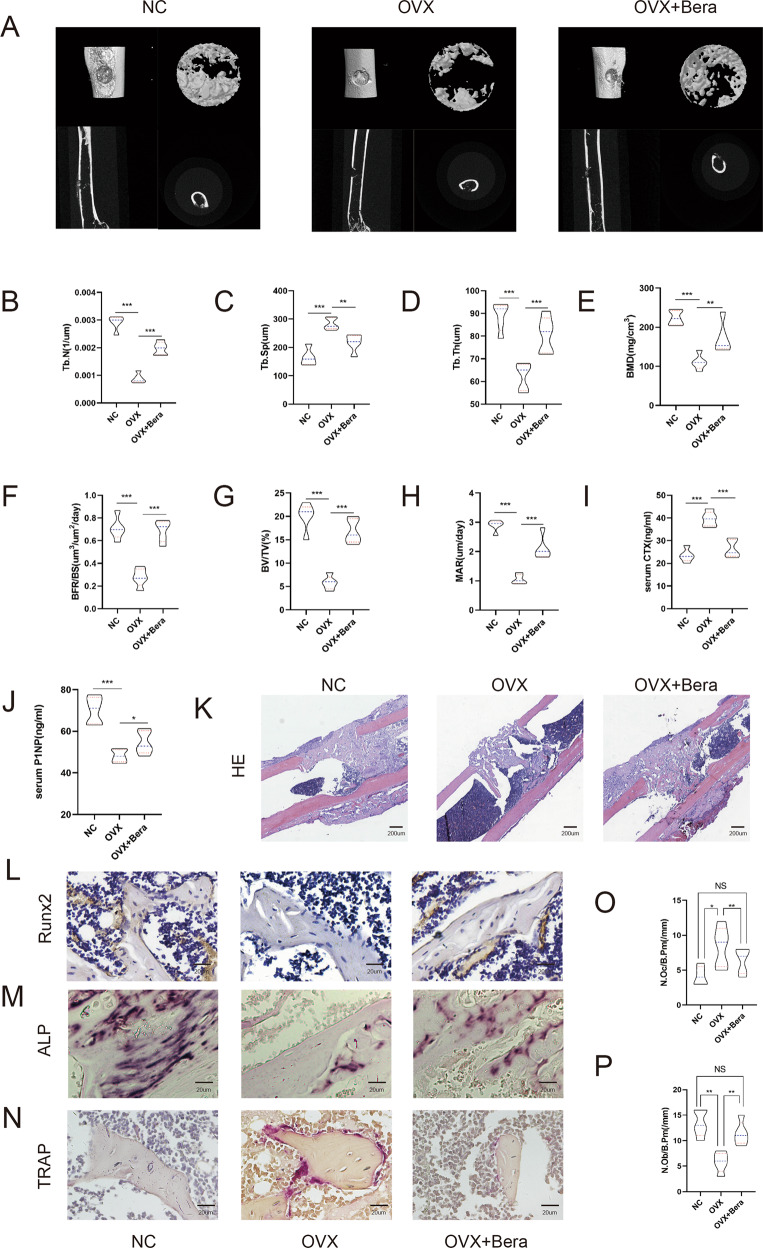
Beraprost promotes the regeneration of bone defects. **A** Representative images of micro-CT reconstruction of femoral cortical bone defects. **B–D** Histomorphometric analysis of the regenerated bone. The mean values of the bilateral defects of each mouse were counted as individual data points. Results are shown as mean ± SD; *n* = 5; **P < 0.01, ***P < 0.001 by ANOVA with Tukey’s post-hoc test. **E**–**H** Quantitative analyses of micro-CT of distal femurs. Results are shown as mean ± SD; *n* = 5; **P < 0.01, ***P < 0.001 by ANOVA with Tukey’s post-hoc test. **I**, **J** Serum concentration of β-CTX and P1NP. Results are shown as mean ± SD; *n* = 5; *P < 0.05, ***P < 0.001 by ANOVA with Tukey’s post-hoc test. **K** H&E staining of femoral bone defects. **L** Representative images of immunohistochemical staining of Runx2. Scale bar, 20 µm. **M**, **N** Representative images of ALP and TRAP staining. Scale bar, 20 µm. **O**, **P** Quantification of Alp and TRAP staining. Results are shown as mean ± SD; *n* = 5, *P < 0.05, **P < 0.01 by ANOVA with Tukey’s post-hoc test.

Next, we studied the function of beraprost in a bone regeneration model of femoral cortical bone defects^25^. Mice were administered beraprost or vehicle (saline) daily by intragastric route for 5 weeks. The femurs were examined one week after the surgery. Consistent with the results of the osteoporosis group model, micro-CT and histological analyses showed that the cortical gaps were nearly fully bridged in mice treated with beraprost (Fig. 2A). The Tb.Th and Tb.N of the mineralized callus in the beraprost group were notably higher than that in the OVX group while the Tb.Sp was lower than that in the OVX group (Fig. 2B–D). The BMD, BFR/BS, BV/TV, and mineral apposition rate (MAR) were clearly increased compared to the OVX group in the bone defect models. (Fig. 2E–H). The concentration of P1NP was higher in beraprost-treated mice than in OVX mice, while the serum concentration of β-CTX decreased (Fig. 2I, J). Immunohistochemical staining showed stronger Runx2 signals in the group treated with beraprost, which was consistent with the results of alkaline phosphatase (ALP) staining (Fig. 2L, M). The signals of tartrate-resistant acid phosphatase (TRAP) staining were weaker in the group treated with beraprost than that in the OVX group (Fig. 2N). Furthermore, histomorphometric analysis revealed a strong increase in osteoblast number/bone perimeter (N.Ob/B.Pm), and a clear decrease in osteoclast number/bone perimeter (N.Oc/B.Pm) in mice administered beraprost compared to that in the OVX group without beraprost (Fig. 2O, P). N.Ob/B.pm and N.Oc/B. Pm were manually counted under the microscope. ALP positive and TRAP positive cells were considered as osteoblasts and osteoclasts.

### Beraprost promotes osteoblastogenesis of BMSCs in vitro

**Fig. 3:**
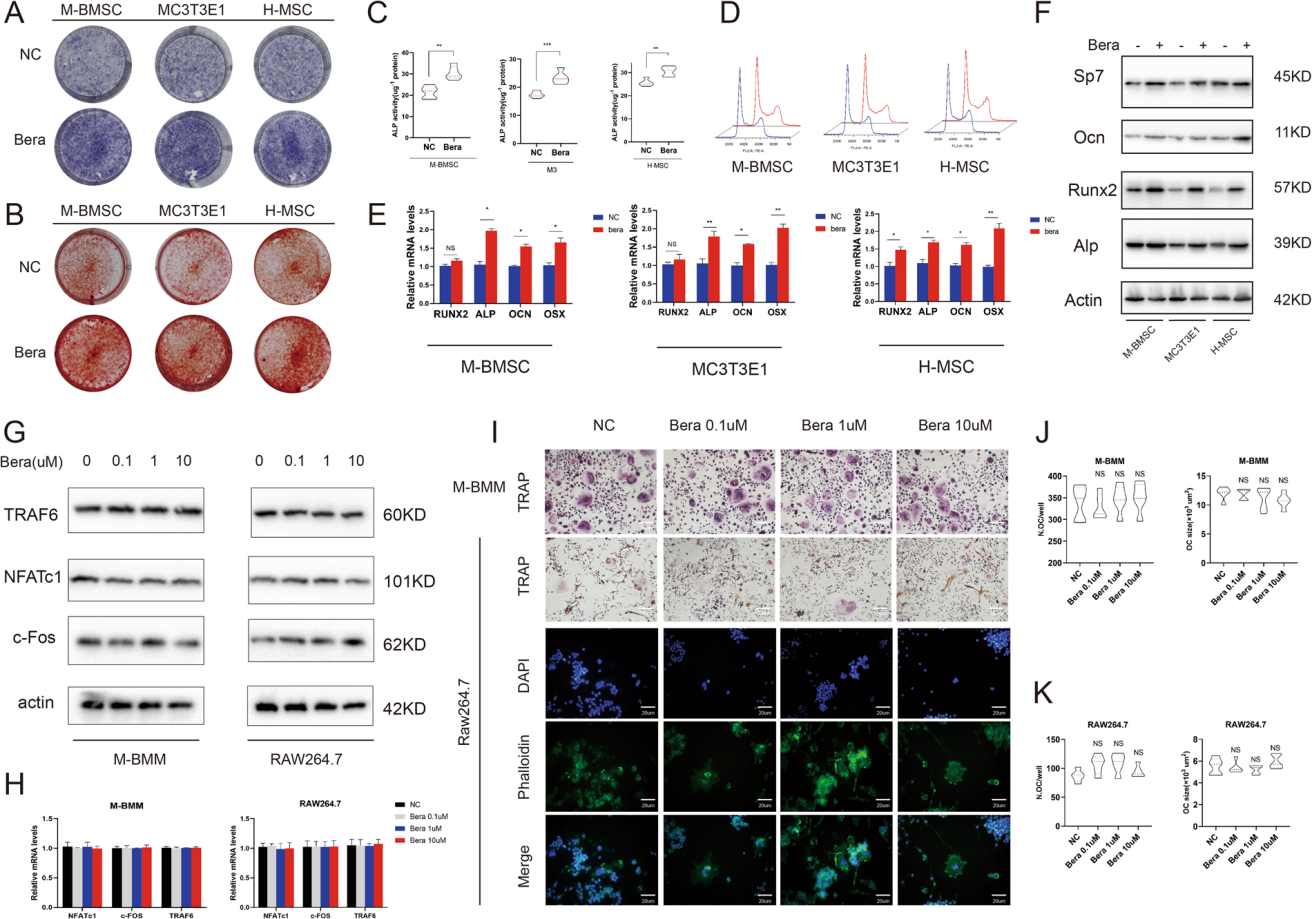
Beraprost promotes osteoblastogenesis but has no direct effect on the differentiation of osteoclast in vitro. **A**, **B** Representative images of ALP and ARS staining. **C** Quantitative analyses of ALP activity. Results are shown as mean ± SD; *n* = 5, **P < 0.01 and ***P < 0.001 by *t* test. **D** Cell cycle was measured by flow cytometry. **E** Quantitative RT-PCR analysis of mRNA expression of Runx2, Alp, OCN, and OSX in beraprost-treated BMSCs. Results are shown as mean ± SD; *n* = 5; *P < 0.05, **P < 0.01, and ***P < 0.001 by *t* test. **F** Western blotting images showing protein expression of Runx2, ALP, OCN, and OSX in beraprost-treated BMSCs. **G** Western blotting images showing protein expression of TRAF6, NFATc1, and c-Fos in beraprost-treated M-BMM and Raw264.7 cells. **H** Quantitative RT-PCR analysis of mRNA expression of TRAF6, NFATc1, and c-Fos in beraprost-treated M-BMM and Raw264.7 cells. Results are shown as mean ± SD; *n* = 5, ns (*P* > 0.05) by ANOVA with Tukey’s post-hoc test. **I** Representative images of TRAP staining of M-BMM and Raw264.7 after 7 days of osteoclast differentiation. Scale bar, 50 µm. Representative images of fluorescent staining of Raw264.7 cells are shown. The cytoskeleton was stained with FITC-conjugated phalloidin and the nucleus was stained with DAPI. RANKL (30 ng/mL) was used in all groups. Scale bar, 20 µm. **J**, **K** Number and size of osteoclasts. TRAP-positive cells with at least three nuclei were defined as osteoclasts. *n* = 5; ns (*P* > 0.05) by ANOVA.

We then evaluated the effect of beraprost on osteoblastogenesis in vitro. M-BMSCs and MC3T3-E1 cells were cultured in osteogenic differentiation medium with or without beraprost. Beraprost notably promoted the osteogenic potential of M-BMSCs, MC3T3-E1 cells, and human MSCs (H-MSCs), as demonstrated by ALP and Alizarin Red S staining (ARS) (Fig. 3A, B). Quantitative analyses confirmed an increase in ALP activity, an early marker of osteogenesis (Fig. 3C). Flow cytometry showed that treatment with beraprost had no significant influence on cell cycle progression (Fig. 3D). More importantly, mRNAs for markers of osteoblastogenesis, including ALP, osterix (OSX) also known as SP7, and OCN, were highly expressed in beraprost-treated BMSCs compared to controls, whereas changes in the expression of Runx2 were not obvious (Fig. 3E). Western blotting showed that beraprost increased the protein levels of the osteoblast differentiation markers (Fig. 3F).

### Beraprost inhibits osteoclast differentiation through BMSCs

**Fig. 4:**
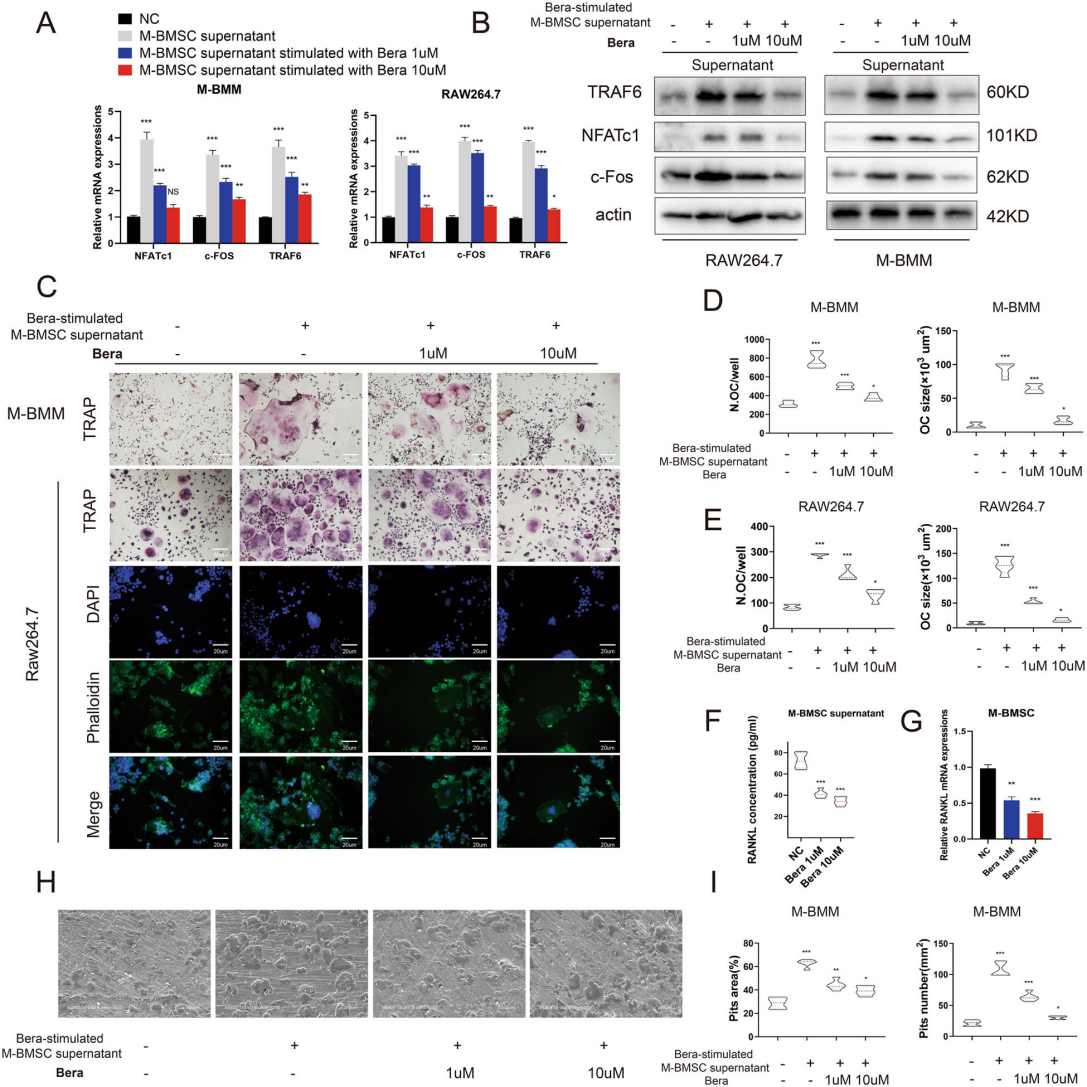
Beraprost promotes osteoclast differentiation through BMSCs in vitro. **A** Quantitative RT-PCR analysis of mRNA expression of TRAF6, NFATc1, and c-Fos in M-BMM and Raw264.7 cells cultured with the supernatant of beraprost-stimulated M-BMSCs. Results are shown as mean ± SD. *n* = 5; *P < 0.05, **P < 0.01, and ***P < 0.001 by ANOVA with Tukey’s post-hoc test. **B** Western blotting images showing protein expression of TRAF6, NFATc1, and c-Fos in M-BMM and Raw264.7 cells cultured with the supernatant of beraprost-stimulated M-BMSCs. **C** Representative images of TRAP staining in M-BMM and Raw264.7 after 7 days of osteoclast differentiation in the presence of M-BMSCs supernatant stimulated with beraprost. Scale bar, 50 µm. Representative images of fluorescent staining of Raw264.7 cells are shown. The cytoskeleton was stained with FITC-conjugated phalloidin and the nucleus was stained with DAPI. RANKL (30 ng/mL) was used in all groups. Scale bar, 20 µm. **D**, **E** Number and size of osteoclasts. TRAP-positive cells with at least three nuclei were defined as osteoclasts. *n* = 5; *P < 0.05, **P < 0.01, and ***P < 0.001 by ANOVA with Tukey’s post-hoc test. **F** Concentration of RANKL in the supernatant. *n* = 5; ***P < 0.001 by ANOVA with Tukey’s post-hoc test. **G** Quantitative RT-PCR analysis of mRNA expression of RANKL in M-BMSCs treated with beraprost. *n* = 5; **P < 0.01, ***P < 0.001 by ANOVA with Tukey’s post-hoc test. **H** Representative images of bone resorption pits captured by scanning electron microscope. Scale bar, 200 µm. **I** Quantitative analyses of pit number and area. Results are shown as mean ± SD; *n* = 5; *P < 0.05, **P < 0.01, and ***P < 0.001 by ANOVA with Tukey’s post-hoc test.

We further studied the direct effect of beraprost on the differentiation of Raw264.7 and mouse bone marrow-derived macrophage (M-BMM). The mRNA and protein expression of osteoclast-related genes, including TRAF6, NFATc1, and c-Fos, did not show obvious changes following stimulation with beraprost (Fig. 3G, H). Osteoclastogenesis was not notably affected by beraprost in the presence of RANKL and macrophage colony stimulating factor (M-CSF), as evidenced by TRAP and fluorescent staining (Fig. 3I–K). RANKL secreted by BMSCs plays an important role in osteoclast differentiation^26,27^. Next, we extracted the supernatant of M-BMSCs and concentrated them in a freeze drier. The lyophilized powder was resuspended in the culture medium to achieve the desired concentration. Interestingly, addition of BMSC supernatant to Raw264.7 or M-BMM cells caused a significant increase in the expression of key osteoclast differentiation genes, such as NFATc1, c-FOS, and TRAF6. Then, we re-collected the supernatant of BMSCs stimulated with a higher concentration of beraprost. The expression of NFATc1, c-FOS, and TRAF6 decreased significantly when the concentration of beraprost was increased further (Fig. 4A, B). The differentiation potential of M-BMMs and Raw264.7 cells was substantially inhibited when they were co-cultured with the supernatant of beraprost-stimulated M-BMSCs compared to that from the group without beraprost stimulation, as demonstrated by TRAP and fluorescent staining (Fig. 4C). The presence of beraprost clearly decreased the number and size of TRAP-positive cells (Fig. 4D, E). ELISA showed decreased levels of RANKL protein in the supernatant of M-BMSCs as the concentration of beraprost was increased (Fig. 4F). Consistent with the ELISA results, the mRNA expression of RANKL was also decreased in the M-BMSCs (Fig. 4G). Electron microscopy showed that M-BMMs cultured with the supernatant from beraprost-stimulated M-BMSCs had fewer resorption pits and a smaller area of resorption overall, indicating a weaker bone absorption capacity (Fig. 4H, I).

### p53 is the key to Beraprost-mediated effects on bone mass

**Fig. 5:**
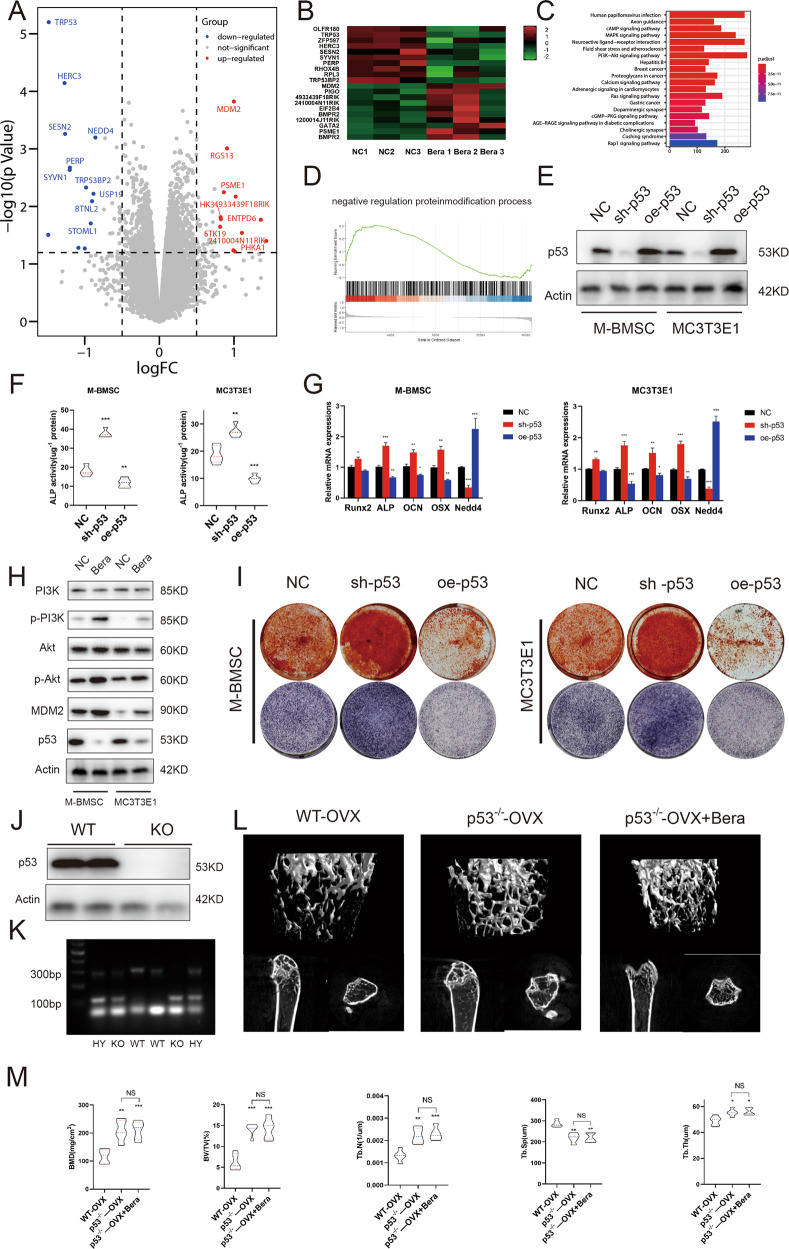
P53 is the key to beraprost-mediated effect on bone mass. **A**, **B** Volcano plots and heatmap of genes with significant changes in beraprost-stimulated M-BMSCs. **C** KEGG pathway analysis indicating altered function of the PI3K–AKT pathway. **D** GSEA showing that processes involved in negative regulation of protein modification were affected. **E** Western blot analysis of the transfection efficiency of p53 in M-BMSCs. **F** Quantitative analysis of ALP activity. *n* = 5; **P < 0.01, ***P < 0.001 by ANOVA with Tukey’s post-hoc test. **G** Quantitative RT-PCR of mRNA expression of Runx2, Alp, OCN, OSX, and Nedd4 in M-BMSCs. Results are shown as mean ± SD; *n* = 5; *P < 0.05, **P < 0.01, and ***P < 0.001 by ANOVA with Tukey’s post-hoc test. **H** Western blotting images of protein expression of pI3k, p-pI3k, AKT, p-AKT, mdm2, and p53 in M-BMSCs. **I** Representative images of ALP and ARS staining. **J** Western blot images showing protein expression of p53 in WT and p53 knockout mice. **K** Images of gene identification by DNA electrophoresis. **L** Representative images of micro-CT reconstruction of distal femurs. **M** Histomorphometric analysis of the distal femurs. Results are shown as mean ± SD; *n* = 5; *P < 0.05, **P < 0.01, and ***P < 0.001 by ANOVA with Tukey’s post-hoc test.

The molecular mechanism of beraprost-mediated effects was elucidated by performing microarray analysis (Fig. 5A, B). Beraprost treatment led to decreased expression of several genes, including p53, an important transcriptional factor involved in cell proliferation (Fig. 5A). Further, KEGG pathway analysis showed that beraprost upregulated the expression of genes related to the PI3K/AKT pathway, which was also confirmed by western blotting (Fig. 5C).

Gene set enrichment analysis (GSEA) showed that beraprost treatment increased the expression of genes involved in negative regulation of the protein modification process in MSCs (Fig. 5D). M-BMSCs and MC3T3-E1 cells transduced with short hairpin RNA (shRNA) lentivirus constructs targeting mouse p53 or lentivirus encoding mouse p53 were cultured in osteoblast differentiation media. The efficiency of transfection was determined by western blotting (Fig. 5E) and the expression of ALP, OSX, OCN, and Runx2, the characteristic osteogenic marker genes^28–30^, was analyzed. Knockdown of p53 promoted osteoblast differentiation, as demonstrated by the increased expression of ALP, OSX, and OCN with Runx2 showing the least significant change (Fig. 5G). Consistent with the results of KEGG analysis, the protein expression levels of p-PI3K, p-Akt, and mdm2 were significantly increased following beraprost treatment. In contrast, the protein expression level of p53 decreased (Fig. 5H).

More interestingly, we found that the expression of Nedd4 was positively related to p53 protein levels. We further probed whether this effect was dependent on p53 protein levels using p53 shRNA or adenovirus encoding p53. Depletion of p53 reduced the mRNA expression of Nedd4, while overexpression of p53 showed the opposite effect (Fig. 5G). Consistent with enhanced osteoblast differentiation, ALP activity and staining was markedly increased in p53 knockdown cells (Fig. 5F, I). Alizarin red staining showed increased mineralization in p53 knockdown cells. Furthermore, overexpression of mouse p53 reversed the effect of p53 knockdown on ALP activity and mineralization capacity, confirming the specificity of the p53 knockdown effect in promoting osteoblast differentiation of M-BMSCs and MC3T3-E1 cells in vitro (Fig. 5I).

These results suggest that p53 may be a vital transcription factor in beraprost-mediated bone mass increase. To verify our hypothesis, we developed OVX models using young female wild-type C57 (WT) and p53 knockout mice. P53 was abrogated in p53−/− mice, as demonstrated by western blotting and electrophoresis (Fig. 5J, K). One group of p53 knockout mice was administered beraprost, while the other group of p53 knockout mice or WT mice were administered saline as a vehicle. BMD as assessed by micro-CT was higher in p53−/− mice than that in WT mice littermates, whereas intragastric administration of beraprost did not cause improvement compared to the p53−/− mice. The BMD, Tb.N, and Tb.Th were increased in p53−/− mice compared to WT mice, whereas p53 knockout abrogated beraprost-mediated bone mass increase (Fig. 5L, M).

### p53 exerts its effects on bone mass by promoting Nedd4 promoter activity

Nedd4 is an important member of the HECT domain E3 ligase family and is conserved among different species^31^. Nedd4 functions by ubiquitinating its substrates and Nedd4 can also alter protein trafficking. We first demonstrated that the suppression of p53 inhibited the expression of Nedd4, whereas overexpression of p53 upregulated the expression of Nedd4. To study the mechanism by which p53 regulates bone metabolism, we carried out a ChIP assay and found that p53 binds to the promoter region of Nedd4.

**Fig. 6:**
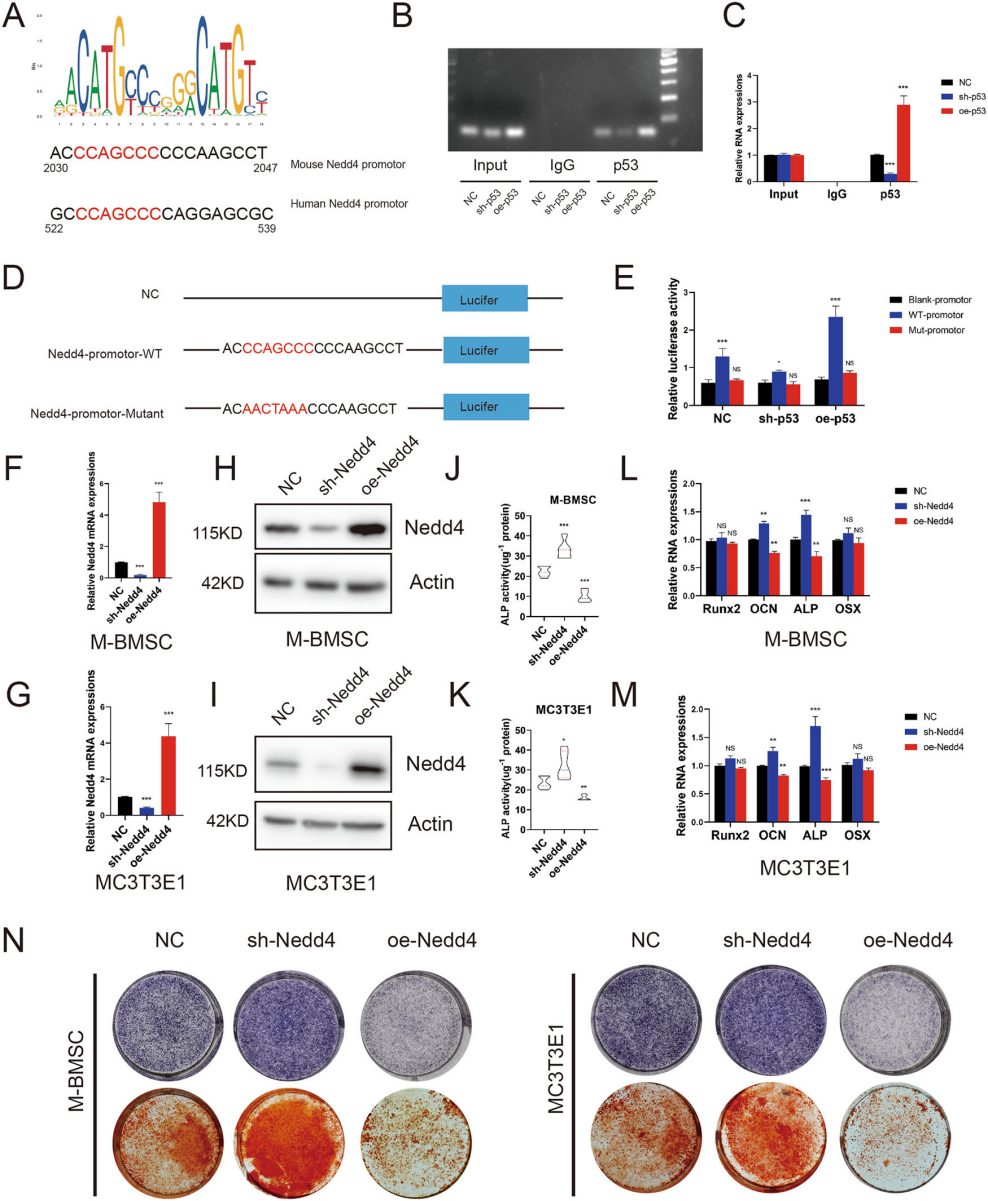
P53 exerts its effects on bone mass by promoting Nedd4 promoter activity. **A** Schematic illustration of the p53-binding sequence and the promoter region of Nedd4 in mice and humans. **B**, **C** P53 binds to its binding sites in the Nedd4 promoter, as shown by the ChIP assay. Results are shown as mean ± SD; *n* = 5; ***P < 0.001 by analysis of variance (ANOVA) with Tukey’s post-hoc test. **D** Mutations in the putative p53-binding site in the Nedd4 promoter region. **E** Mutations in the binding site decreased luciferase activity in M-BMSCs. Results are shown as mean ± SD; *n* = 5; *P < 0.05, **P < 0.01, and ***P < 0.001 by ANOVA with Tukey’s post-hoc test. **F–I** qRT-PCR and western blotting were used to measure the transfection efficiency of Nedd4 in M-BMSCs and MC3T3-E1 cells. *n* = 5; ***P < 0.001 by ANOVA with Tukey’s post-hoc test. **J**, **K** Quantitative analysis of ALP activity. *n* = 5; *P < 0.05, **P < 0.01, and ***P < 0.001 by ANOVA with Tukey’s post-hoc test. **L**, **M** qRT-PCR showing mRNA expression of Runx2, Alp, OCN, and OSX in M-BMSCs. Results are shown as mean ± SD; *n* = 5; ns (*P* > 0.05), **P < 0.01 and ***P < 0.001 by ANOVA with Tukey’s post-hoc test. **N** Representative images of ALP and ARS staining.

We predicted p53-binding sites within the Nedd4 promoter regions in JASPAR (http://jaspar.genereg.net). Furthermore, to confirm that p53 regulates Nedd4 transcription, we performed ChIP assay in M-BMSCs (Fig. 6A, B). Interestingly, overexpression of p53 caused increased binding of p53 to the Nedd4 promoter site in M-BMSCs and conversely, whereas knockdown of p53 using shRNA resulted in decreased p53 binding to the Nedd4 promoter site. Next, we quantified the binding of the Nedd4 promoter site by p53 using ChIP-PCR (Fig. 6C). The PCR results were consistent with the DNA electrophoresis results. The promoter was then cloned into a luciferase reporter plasmid (Fig. 6D). We introduced mutations in the predicted p53-binding site of the Nedd4 promoter and found that these mutations decreased luciferase activity (Fig. 6E).

We further evaluated the osteogenic capacity of M-BMSCs and MC3T3-E1 cells after modulation of expression of Nedd4. We knocked down or overexpressed Nedd4 in M-BMSCs and MC3T3-E1 cells using lentiviruses (Fig. 6F–I). The capacity of the osteogenic cells was measured 14 days after osteogenic induction (Fig. 6J, K). We then measured the mRNA expression of Runx2, Alp, OSX, and OCN. These results illustrated that diminished Nedd4 in M-BMSCs and MC3T3-E1 cells increased the expression of Alp, OSX, and OCN during osteogenic induction; however, overexpression of Nedd4 had the opposite effect (Fig. 6L, M). ALP and ARS staining illustrated that Nedd4 knockdown resulted in more intense staining, while the overexpression of Nedd4 led to less intense staining during osteogenesis (Fig. 6N). In conclusion, these results illustrated that Nedd4 inhibited the osteogenic differentiation potential of M-BMSCs and MC3T3-E1 cells in vitro.

### Nedd4 directly recognizes Runx2

**Fig. 7:**
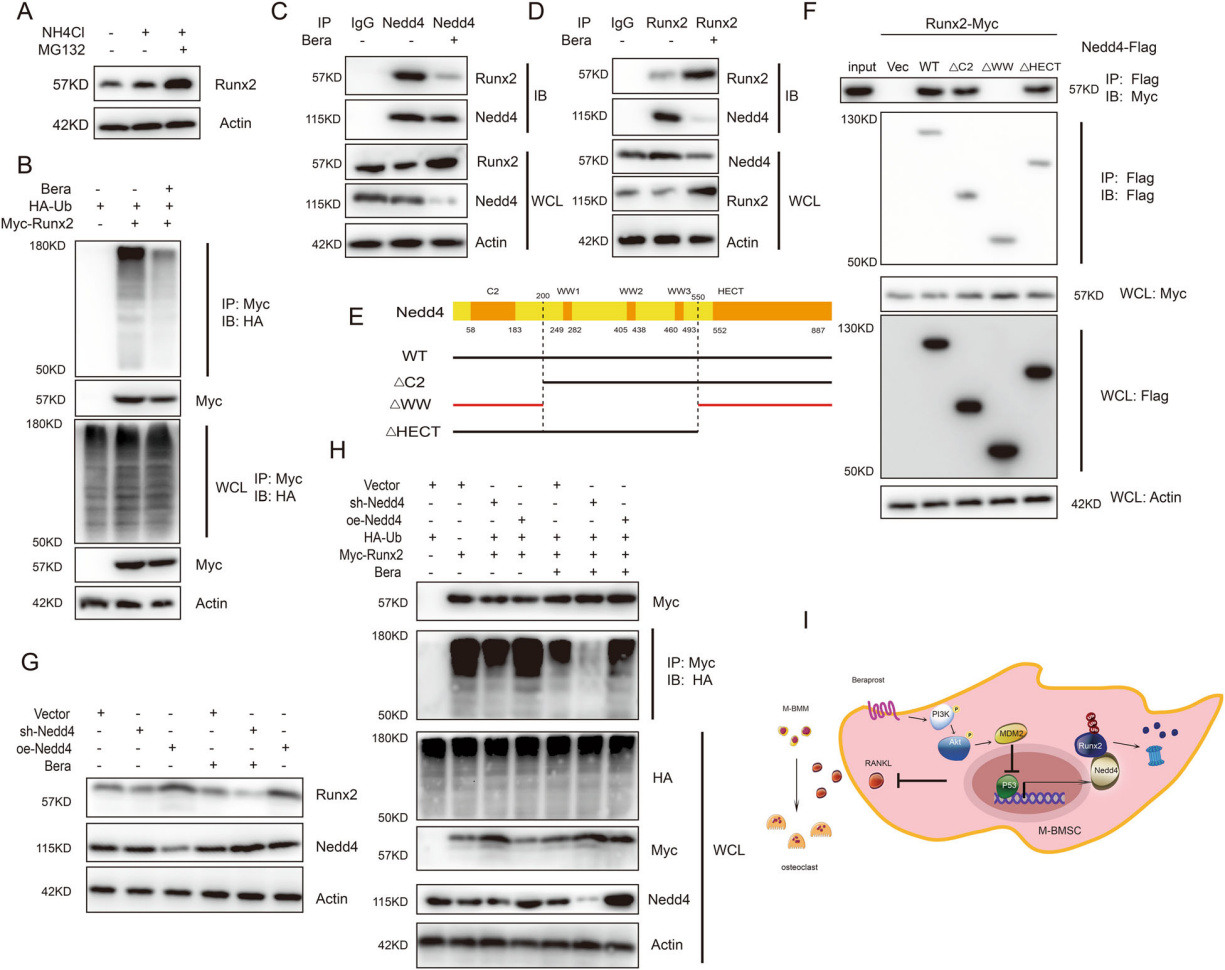
Nedd4 directly recognizes Runx2. **A** M-BMSCs treated with beraprost (10 µM) for 12 h were cultured with MG132 (50 mM) or NH_4_Cl (50 mM) for 6 h, and Runx2 protein level was analyzed by western blotting. **B** M-BMSCs were transfected with the indicated plasmids. Two days after transfection, the cells were treated with PBS or beraprost (10 µM) for 12 h, and then lysed using RIPA buffer. Lysates were immunoprecipitated with mouse anti-myc antibody. Western blotting was performed to analyze the levels of ubiquitination. **C**, **D** Interaction between endogenous Runx2 and Nedd4 following beraprost treatment. M-BMSCs treated with PBS or beraprost (10 µM) for 12 h were lysed with RIPA buffer. Whole-cell lysates (WCL) were used for IP with IgG, anti-Nedd4 antibody or anti-Runx2 antibody, followed by immunoblotting (IB) with the indicated antibodies. **E** Schematic illustration of wild-type and deletion mutants of Nedd4. **F** The WW domain of Nedd4 is necessary for interaction with Runx2. Myc-Runx2 was co-transfected with various flag-Nedd4 deletion mutants in M-BMSCs. WCLs were used for immunoprecipitation with anti-flag antibody, and IB with anti-flag or anti-myc antibodies. WCL was IB as a control to show the protein expression. **G** M-BMSCs transfected with lentiviral vector, lentiviral shRNA against Nedd4 or lentivirus encoding Nedd4 were treated with PBS or beraprost (10 µM) for 12 h. Protein expression levels of Runx2 and Nedd4 were measured by western blotting. **H** Knockdown of Nedd4 decreased the ubiquitination of Runx2 in vitro. M-BMSCs were transfected with the indicated plasmids. Two days after transfection, the cells were treated with PBS or beraprost (10 µM) for 12 h, and the cells were lysed with RIPA buffer. Lysates were immunoprecipitated with mouse anti-myc antibody. Western blotting was performed to analyze the levels of ubiquitination. **I** Schematic illustration of the role of beraprost in postmenopausal osteoporosis.

As shown above, beraprost upregulated the expression of Runx2 mainly at the protein level and not at the mRNA level. While GSEA showed increased expression of genes involved in negative regulation protein modification process in beraprost-treated M-BMSCs, we hypothesized that Nedd4 mediates the degradation of Runx2 protein. To verify our hypothesis, cells were treated with either NH_4_Cl (a lysosome inhibitor) or MG132 (a proteasome inhibitor). We found that Runx2 protein was more sensitive to MG132, confirming that the degradation of Runx2 occurred mainly through the proteasome pathway (Fig. 7A). Furthermore, we found that beraprost treatment decreased the ubiquitination of myc-Runx2 (Fig. 7B). In conclusion, our data indicate that beraprost inhibits ubiquitin–proteasomal degradation of Runx2 in M-BMSCs.

Next, we predicted Nedd4 as a primary E3 ligase for Runx2 in the UbiBrowser database (http://ubibrowser.ncpsb.org/). Co-immunoprecipitation (co-IP) assays were performed to confirm the interaction between endogenous Runx2 and Nedd4. A weak interaction was found following beraprost treatment, suggesting that Nedd4 interacted with Runx2 in M-BMSCs (Fig. 7C, D). We further found that the WW domain of Nedd4 was essential for Runx2 binding (Fig. 7E). Knockout of the WW domain resulted in the inability of Nedd4 to bind to Runx2 (Fig. 7F). To study whether expression of Nedd4 mediated the beraprost-inhibited degradation of Runx2, shRNA lentiviral directed against Nedd4 or lentiviral encoding Nedd4 were transfected into M-BMSCs. We found that decrease in Nedd4 resulted in a marked increase in Runx2, and this effect was more evident following beraprost treatment. In contrast, overexpression of Nedd4 resulted in a decrease in Runx2 levels (Fig. 7G). We found that knockdown of Nedd4 notably reduced the ubiquitination of Runx2 in M-BMSCs, whereas overexpression of Nedd4 showed the opposite effect, which was consistent with our previous observations (Fig. 7H).

## Discussion

Osteoporosis is a common age-related disease. Fractures caused by osteoporosis and increase in population aging have inspired researchers to continue to identify novel anti-osteoporosis factors^32^. So far, the drugs used for osteoporosis in clinical practice are mainly divided into two types: agents that promote bone formation and bone resorption inhibitors. Misoprostol, a prostaglandin E2 analog, has been reported to have an obvious effect on improving bone mass in postmenopausal women in a clinical study, although the underlying mechanism has not been elucidated. Therefore, we focused on a prostaglandin analog, beraprost. Beraprost has been reported to enhance the APC function of B cells^33^. In addition, McKean first reported that beraprost inhibits human vascular smooth muscle cell migration via an exchange protein directly activated by cAMP^34^. However, the role of beraprost in osteoporosis remains unclear. We first reported that beraprost treatment increases bone mass resulting from the inhibition of bone resorption and the promotion of bone formation.

We then showed that supplementation with beraprost not only promoted the healing of bone defects, but also prevented the loss of bone mass in OVX mice. Immunohistochemistry (IHC) and trap staining indicated that this effect may be related to the promotion of osteoblast differentiation and inhibition of osteoclast maturation in vivo. As a functional protein in osteoblast differentiation, Runx2 has been significantly increased after beraprost treatment. We then investigated whether beraprost plays a similar role in vitro. In response to beraprost, the expression levels of osteogenic differentiation-related proteins in M-BMSCs and MC3T3-E1 cells were significantly increased. To rule out that the changes in ALP and ARS staining were caused by the effect of beraprost on cell proliferation, we performed cell cycle detection using flow cytometry. Furthermore, we found that beraprost had no direct effect on osteoclast differentiation. Beraprost inhibits osteoclastogenesis by reducing the secretion of RANKL by BMSCs.

To investigate the underlying mechanism, we performed RNA-Seq assay and identified p53 as the gene that changed most significantly following beraprost treatment. p53 plays a critical role in bone remodeling^23,35^. Mice with p53 gene knockout had higher bone mass than controls. Wang et al. reported that p53 functions as a negative regulator of osteoblastogenesis^22^. Jin et al. reported that Bre enhances osteoblast differentiation by promoting Mdm2-mediated degradation of p53^36^. These findings suggest that p53 promotes loss of bone mass. KEGG pathway analysis indicated that the PI3K–AKT pathway is related to the functions of beraprost. We found that the regulation of p53 gene expression directly affected the differentiation ability of osteoblasts. Furthermore, the effect of beraprost in promoting bone mass increase was greatly limited in p53 knockout mice. P53 acts as an important tumor suppressor gene. The decrease of p53 may increase the risk of cancer. Our study shows that beraprost inhibits p53 in BMSCs. Whether beraprost can exert a similar effect in other cells is still unknown. At present, we have not found the occurrence of cancer in mice after taking beraprost for 3 months.

We verified the sequencing results by PCR and found that the gene expression of Nedd4 also decreased significantly following beraprost treatment. As a tumor suppressor gene, p53 is considered to be a critical transcription factor that regulates gene transcription. Given that the changes in Nedd4 and p53 expression were positively correlated, we hypothesized that p53 acts as a transcription factor of Nedd4 to regulate its transcription. Using promo software (http://alggen.lsi.upc.es/), we predicted a p53-binding site in the Nedd4 promoter region that was conserved among species. Mutation of the binding site caused a rapid reduction in luciferase activity indicating that the affinity of p53 binding to the promoter of Nedd4 was reduced.

Nedd4 is a ubiquitin E3 ligase^37^. It is comprised of a catalytic C-terminal HECT domain, a N-terminal calcium/lipid-binding domain (C2 domain), and WW domains responsible for cellular localization and substrate recognition^31,38^. Nedd4 exerts its molecular function mainly by ubiquitinating its substrates^39,40^. Ubiquitination of osteogenic-related proteins is closely related to bone growth and remodeling^41,42^. Zou et al. reported that the E3 ubiquitin ligase WWP2 regulates craniofacial development through mono-ubiquitylation of goosecoid^43^. Zhu et al. reported that WWP2 catalyzes the mono-ubiquitination of Runx2, a member of the Nedd4 family of E3 ubiquitin ligases^44^. Kaneki et al. showed that Runx2 was degraded by Smurf1 and Smurf2^45^. Jiang et al. found that Nedd4 inhibits the osteogenic differentiation of BMSCs through the p38 MAPK pathway^46^.

The promoter region for ubiquitin E3 ligases such as smurf1/2 may be regulated by P53 as they have similar WW domains as Nedd4. However, variance analysis of RNA-sequencing suggested that Nedd4 showed the most obvious decline among ubiquitin E3 ligases after beraprost treatment (Fig. 5A). Base on the analysis of the RNA-sequencing results, the transcription-promoting activity of p53 on these ubiquitin E3 ligases may be weaker than that of Nedd4 under the assumption that p53 can bind to the promoter region of these ubiquitin E3 ligases. Therefore, we mainly put our attention on Nedd4.

As GSEA indicated the involvement of the protein modification process, we hypothesized that Nedd4 plays an essential role in the process of beraprost-mediated regulation of osteogenesis. As we have demonstrated, although Runx2 protein level increased significantly after beraprost treatment, increase in its mRNA expression was not consistent. Further, Runx2 protein level was not significantly affected by beraprost following p53 knockdown when the expression of Nedd4 was low (Fig. S). We then used the Unibrowser software (http://ubibrowser.ncpsb.org/) to predict the interaction of Nedd4 with Runx2, and verified the interaction between of the WW domain of Nedd4 and Runx2 by co-IP assay.

Beraprost is widely used for the treatment of arterial occlusions. Patients who had received beraprost treatment over an extended period of time were screened out, and their bone metabolism indexes in the serum were tested. Few of the bone metabolism indicators were slightly increased, which suggests that beraprost may have potential value in the treatment of osteoporosis.

## Materials and methods

### Cell culture

M-BMSCs, MC3T3-E1 cells (ATCC), and H-MSCs were cultured in Dulbecco’s modified Eagle’s medium (DMEM, # SH30022; Hyclone, USA) containing 10% fetal bovine serum (Thermo Fisher, Waltham, MA, USA), 100 U/mL of penicillin (Invitrogen, USA), and 100 μg/mL of streptomycin (Invitrogen, USA) under 5% CO_2_ at 37 °C in a humidified atmosphere, whereas Raw264.7 cells (ATCC) and M-BMMs were cultured in α-MEM (#SH30265; Hyclone, USA) containing 10% fetal bovine serum, 100 U/mL of penicillin, and 100 μg/mL of streptomycin.

### IHC and immunofluorescence (IF) staining

Bone tissue sections were fixed in 10% buffered formalin and then embedded in paraffin. Slides were deparaffinized in xylene before rehydration with alcohol for IHC and IF staining. Hydrogen peroxide (3%) was used to block endogenous peroxidase for IHC. For antigen retrieval, 0.1 M citric sodium buffer was used under microwave irradiation. The sections were blocked with 5% BSA for 30 min and then incubated with the primary antibody overnight at 4 °C. After washing thrice with phosphate buffered saline (PBS), antibody binding was detected with an HRP-DAB kit (KIT-5920, Maxvision^TM^2 HRP-Polymer anti-Mouse/Rabbit IHC Kit) and counterstained with hematoxylin. For IF, a secondary antibody with a fluorescent label was used to bind to the primary antibody. Images were acquired using an Olympus microscope (Olympus BX51).

### Chromatin immunoprecipitation (ChIP) analysis

In brief, using a ChIP kit (P2078, beyotime), cells were crosslinked with 1% formaldehyde by incubating for 10 min at 37 °C and the reaction was stopped with glycine. After washing twice with cold PBS, the crosslinked cells were collected, spun and washed twice with PBS. The cell pellet was then resuspended in lysis buffer supplemented with phenylmethanesulfonyl fluoride (PMSF) for 10 min on ice. Then, the lysates were sonicated (Sonics, amplitude 25%, 8 cycles of 10 s on and 10 s off mode) into 100–300 bp DNA fragments. The debris was pelleted by centrifugation at 20,000 × *g* at 4 °C for 5 min, and the supernatant was transferred to new tubes. The chromatin samples were then diluted in cold dilution buffer containing PMSF. Protein A + G agarose/salmon sperm DNA was added and incubated for 60 min to reduce non-specific binding. Then, the samples were cleared by centrifugation and the supernatant was transferred to sterile tubes. A total of 10 µL of the supernatant was used as the input and incubated with the primary antibody at 4 °C overnight with slow rotation. After incubation, protein A + G agarose/salmon sperm DNA was added to the supernatant and rotated slowly at 4 °C for 60 min. After centrifugation (3000–5000 × *g*, 1 min), the protein A + G agarose was precipitated and the supernatant was removed. The precipitate was then washed with buffer, slowly rotated, and centrifuged at 1000 × *g* for 1 min at 4 °C. An elution buffer (EB) was then added and incubated for 15 min at room temperature followed by a brief centrifugation (3000–5000 × *g*, 1 min). The precipitate was collected and transferred to a new tube and after washing the precipitate with EB, the supernatant was collected. NaCl was used to de-crosslink the DNA-protein complexes. After incubation with ribonuclease A at 37 °C for 30 min, EDTA, Tris–HCl, and proteinase K were added and incubated at 45 °C for 1 h. The purified DNA was then prepared for further use.

### Animals

All animal studies were approved by the Ethics Committee of the Xinhua Hospital affiliated to the Shanghai Jiao Tong University School of Medicine. Female C57BL mice (10 weeks old, weighing 20–25 g) were purchased from the Shanghai Jihui Laboratory Animal Care Co. Ltd. (Shanghai, China) and housed in pathogen-free facilities under a 12-h light and 12-h dark cycle. The p53 knockout mice were purchased from the Jackson Laboratory (B6.129S2-Trp53tm1Tyj/J). Mice were grouped by random number. First, 15 mice were randomly divided into three groups (*n* = 5 per group) and used to check the effect of beraprost on bone mass. Five mice underwent sham surgery while the others underwent OVX. Next, 15 female mice randomly divided into three groups (*n* = 5 per group) and used for bone defect assays. At last, 5 wild-type mice and 10 p53 knockout mice were used for the OVX models.

### Micro-CT analysis and calcein double-labeling analysis

Mice were injected with calcein (10 mg/kg) 8 and 3 days before sacrifice. After sacrifice, the femurs were excised from the experimental mice, immediately fixed with 70% ethanol for 24 h and scanned with a Scanco µCT 40 scanner (Scanco Medical AG, Zurich, Switzerland) at a resolution of 18 µm. The region of interest (ROI) was set as the trabecular bone 1 mm beneath the epiphyseal growth plate. Then, the bone samples were processed sequentially using 90% ethanol, 100% ethanol, LR white hydrophilic medium, and then heated at 60 °C for 12 h. Finally, 5 µm-thick sections were obtained using a LEICA SP1600 Saw Microtome and imaged under fluorescence microscopy for calcein double-labeling analysis (Olympus BX51).

### ELISA

ELISA was used for the quantitative analysis of P1NP, β-CTX, and RANKL. Concentrations of β-CTX and P1NP in the serum or that of RANKL in the supernatant were measured using ELISA kits (ELISAGenie). All procedures were performed according to the manufacturer’s guidelines. Blood was collected 5 weeks after beraprost treatment and the mice were fasted for 6 h prior to the blood collection.

### ALP and mineralization assay

Primary M-BMSCs were obtained from the bone marrow of 4-week-old female C57BL/6 mice. The cell suspension obtained by flushing the bone marrow was cultured in DMEM with 1% penicillin/streptomycin and 10% FBS. The cells were then passaged at a density of 90% confluence. After the second passage, cells were used for osteoblast differentiation. M-BMSCs, MC3T3-E1 cells, and H-MSCs were seeded in six-well plates at 5000 cells/cm². Cells were cultured with osteogenic differentiation medium (10 mM β-glycerophosphate, 0.1 μM dexamethasone, and 0.05 mM ascorbic acid) supplemented with or without 10 µM beraprost until 80% confluent. The culture medium was replaced every 2 days. ALP and ARS staining were performed on the 7th and 14th day, respectively, of osteogenesis. For ALP staining, cells were fixed with 4% paraformaldehyde and incubated at 37 °C with 0.1 M Tris buffer (pH 9.3) containing 0.25% naphthol AS-BI phosphate and 0.75% Fast Blue BB one week later. ALP activity was detected with an ALP staining kit (Cell Biolab, San Diego, CA, USA). For ARS assays, cells were cultured in differentiation medium for 14 days. After washing twice with PBS, the cells were fixed with 4% paraformaldehyde and stained with 1% Alizarin Red S (pH 4.2, Sigma-Aldrich) for 10 min.

### Surgeries

Ketamine (120 mg/kg, Medistar, Ascheberg, Germany) and xylazine (16 mg/kg, Riemser, Greifswald, Germany) was used to anesthetize the mice. For bilateral OVX, the ovaries were cut through the midline dorsal skin and the muscle layer. Then, the ovaries were removed after ligating the oviduct. The incision was sutured using 4-0 silk sutures. For femoral cortical bone defects, after skin incision and blunt dissection of the quadriceps, the surfaces of the middle femurs were exposed. A 0.9 mm hole was created using a round bur.

### Western blotting

Cells were lysed in radioimmunoprecipitation assay (RIPA) buffer (Beyotime, P0013C) containing PMSF (Beyotime, ST506) on ice. Then, 12% sodium dodecyl sulfate polyacrylamide gel electrophoresis (SDS–PAGE) was used to separate the proteins in the lysates. Proteins were then transferred to PVDF membranes (0.22 μm, Millipore) and blocked with free quick blocking buffer (Epizyme, PS108P) for 15 min at room temperature. The membranes were incubated overnight at 4 °C with primary antibodies and washed thrice with TBST before incubation with horseradish peroxidase (HRP)-conjugated secondary antibodies (Beyotime) at room temperature for 1 h. ECL reagent (Epizyme) was used for the detection of antibody–antigen complexes. Antibodies against Myc (#2272), HA (#3724), Flag (#14793), Runx2 (#12556), TRAF6 (#8028), NFATc1 (#8032), c-FOS (#2250), β-actin (#4970), PI3K (#4257), p-PI3K (#17366), Akt (#4691), p-Akt (#4060), MDM2 (#86934), p53 (#2527), Nedd4 (#5344) were obtained from Cell Signaling Technology (Boston, USA). Antibodies against OSX (#ab209484), OCN (#ab133612) were purchased from Abcam (Cambridge, UK).

### Quantitative RT-PCR

RNA was extracted from the different cells using TRIzol reagent (Invitrogen) according to the manufacturer’s instructions. After lysis with TRIzol, chloroform was added to the samples and the samples were centrifuged at 12,000 × *g* for 15 min. An equal volume of isopropanol was added to the upper transparent RNA-containing solution. After centrifugation, the RNA pellet was washed twice with 75% ethanol. RNA was reverse transcribed into cDNA using PrimeScript™ RT Master Mix (Takara, RR036A). Real-time PCR was performed using HieffTM PCR Master Mix (Yeasen) according to the manufacturer’s instructions, and the relative expression of target genes was normalized to that of GAPDH.

### Lentiviral transduction in vitro

Cells were plated at 25% density before transfection. Lentivirus was transfected into cells in the presence of polybrene (5 μg/mL) in serum-free α-MEM medium. After 24 h of transduction, the virus-containing medium was removed and normal α-MEM medium was added to the cells and cultured. Two days after transfection, puromycin was used for cell selection. Floating cells were removed and adherent cells were considered to be transfected cells.

### TRAP staining

Primary mouse BMMs were obtained from the bone marrow of 4-week-old female C57BL/6 mice. The cell suspension flushed from the bone marrow was then cultured in α-MEM with 10% FBS. M-CSF (30 ng/mL) was added to the culture medium for 3 days to obtain BMMs. Raw264.7 cells or M-BMMs from mouse femurs were cultured in 48-well plates at a concentration of 2 × 10^4^/well. RAW264.7 cells were incubated with 100 ng/mL RANKL with or without beraprost. M-BMMs were incubated with 100 ng/mL RANKL and 30 ng/mL M-CSF with or without beraprost. After 7 days of induction, the cells were washed and fixed with 4% paraformaldehyde for 30 min. The cells were then stained with TRAP using a commercial kit (Sigma-Aldrich). TRAP^+^ cells containing at least three nuclei were identified as osteoclasts. The number of osteoclasts was counted under a microscope (Olympus BX51).

### Sequencing

M-BMSCs were cultured in 10-cm dishes and divided into two groups based on treatment with beraprost or not. Each group contained three replicates. Three days after treatment, total RNA was extracted using TRIzol reagent (Invitrogen). RNA was reverse transcribed using Qiagen’s Quantitect Reverse Transcription Kit (Qiagen, Valencia, CA, USA), and the transcript levels were detected using the Illumina MouseRef-8 BeadChip array (Illumina, San Diego, CA, USA) for microarray studies. Beadstudio (Illumina) was used to pre-process the raw data. Genes that were under-expressed or not expressed (*P* ≤ 0.05) were excluded from the analysis. The limma package was used for data analysis.

### Luciferase reporter assay

M-BMSCs were cultured in a 12-well plate at a density of 2 × 10^4^ cells/well. Wild-type or mutant Nedd4 promotor plasmids were constructed by cloning the cDNA into the pGL3-Basic vector. Renilla-containing phRL-TK plasmid was used to normalize transfection efficiency. Luciferase assay was then carried out using a dual-luciferase reporter assay kit according to the manufacturer’s protocol (Promega) 24 h after transfection.

### Plasmids

Flag-tagged wild-type (wt) Nedd4 and Nedd4ΔC2, Nedd4ΔWW, and Nedd4ΔHECT mutants were constructed by cloning the full-length or truncated mutant cDNAs into the BamHI and XhoI sites of the PGMLV-CMV-MCS-3×flag-PGK-Puro vector. All constructs were confirmed by sequencing.

### Resorption pit assay

The function of osteoclasts was examined using the resorption pit assay. BMMs were seeded at a density of 1 × 10^4^ cells/well onto bovine bone slices in a 96-well plate. The wells without bovine bone slices cultured with BMMs were used to observe the morphology of osteoclasts during the differentiation process. BMMs cultured with the supernatant collected from different groups were treated with 50 ng/mL RANKL and 30 ng/mL M-CSF for 7 days to generate mature osteoclasts, as mentioned above. The cells were brushed off the surface of the bone slices. The slices were then sprayed with gold on the surface. Resorption pits in the slices were photographed under a scanning electron microscope (FEI Instr. Software, Hillsboro, OR, USA).

### Co-IP

After washing twice with PBS, cells were lysed with RIPA buffer at 4 °C for 30 min. The scraped cells were centrifuged at 15,000 × *g* for 15 min at 4 °C, and the supernatant was collected for quantification. Then, the protein samples were incubated with IgG of the same species and protein A + G agarose and slowly rotated at 4 °C for 2 h to remove non-specific-binding proteins. The supernatant was obtained by centrifugation at 1000 × *g* for 5 min and incubated with the primary antibody overnight at 4 °C. Then, protein A + G agarose was used to pull down the antibody complex. Finally, 1× SDS–PAGE electrophoresis loading buffer was added to resuspend the pellet and the sample was boiled for 5 min. The sample was stored at −80 °C or used for electrophoresis.

### Statistical analysis

For statistical analysis, each experiment was performed at least three independent replicates. Data are expressed as mean ± SD. Statistically significant differences were assessed by two-tailed Student’s *t* test for comparison between two groups. One-way analysis of variance followed by Tukey’s test was used for comparisons between multiple groups. Statistical significance was set at *P* < 0.05.

## Supplementary information

supplemental figure

supplemental figure legend

## Data Availability

The microarray data is available at https://share.weiyun.com/LZr6qfyr.

## References

[1] Compston JE, McClung MR, Leslie WD (2019). Osteoporosis. Lancet.

[2] Rachner TD, Khosla S, Hofbauer LC (2011). Osteoporosis: now and the future. Lancet.

[3] Black DM, Rosen CJ (2016). Clinical practice. Postmenopausal osteoporosis. N. Engl. J. Med..

[4] Bartelt A (2018). Lrp1 in osteoblasts controls osteoclast activity and protects against osteoporosis by limiting PDGF-RANKL signaling. Bone Res..

[5] Sims NA, Martin TJ (2014). Coupling the activities of bone formation and resorption: a multitude of signals within the basic multicellular unit. Bonekey Rep..

[6] Tanaka Y, Nakayamada S, Okada Y (2005). Osteoblasts and osteoclasts in bone remodeling and inflammation. Curr. Drug Targets Inflamm. Allergy.

[7] Diab DL, Watts NB (2013). Postmenopausal osteoporosis. Curr. Opin. Endocrinol. Diabetes Obes..

[8] Deftos LJ, Schiff SJ (1995). Predicting PTH pulses and patterns in osteoporosis. J. Clin. Investig..

[9] Zhou S, Huang G, Chen G (2020). Synthesis and biological activities of drugs for the treatment of osteoporosis. Eur. J. Med. Chem..

[10] Barrett-Connor E, Stuenkel CA (2001). Hormone replacement therapy (HRT)–risks and benefits. Int. J. Epidemiol..

[11] Starling S (2021). New anti-osteoporosis drug target identified. Nat. Rev. Endocrinol..

[12] Snyder S (2020). Bisphosphonates for osteopenia in postmenopausal women. JAMA.

[13] Tsourdi E, Rachner TD, Hofbauer LC (2018). Romosozumab versus alendronate and fracture risk in women with osteoporosis. N. Engl. J. Med..

[14] Khosla S, Hofbauer LC (2017). Osteoporosis treatment: recent developments and ongoing challenges. Lancet Diabetes Endocrinol..

[15] Cooper LT (2003). Beraprost for the treatment of intermittent claudication. J. Am. Coll. Cardiol..

[16] Demolis JL, Robert A, Mouren M, Funck-Brentano C, Jaillon P (1993). Pharmacokinetics and platelet antiaggregating effects of beraprost, an oral stable prostacyclin analogue, in healthy volunteers. J. Cardiovasc. Pharmacol..

[17] Yasar L (2006). Effect of misoprostol on bone mineral density in women with postmenopausal osteoporosis. Prostaglandins Other Lipid Mediat..

[18] Bauer DC (2019). Bone turnover markers in osteoporosis-reply. JAMA.

[19] Ma Y (2021). Cadmium exposure triggers osteoporosis in duck via P2X7/PI3K/AKT-mediated osteoblast and osteoclast differentiation. Sci. Total Environ..

[20] Zhang Y (2020). PSMC6 promotes osteoblast apoptosis through inhibiting PI3K/AKT signaling pathway activation in ovariectomy-induced osteoporosis mouse model. J. Cell. Physiol..

[21] Sun X (2019). Protective effects of Dipsacus asper polysaccharide on osteoporosis in vivo by regulating RANKL/RANK/OPG/VEGF and PI3K/Akt/eNOS pathway. Int. J. Biol. Macromol..

[22] Wang X (2006). p53 functions as a negative regulator of osteoblastogenesis, osteoblast-dependent osteoclastogenesis, and bone remodeling. J. Cell Biol..

[23] Yu T (2020). p53 plays a central role in the development of osteoporosis. Aging.

[24] Jewett KA (2016). Feedback modulation of neural network synchrony and seizure susceptibility by Mdm2-p53-Nedd4-2 signaling. Mol. Brain.

[25] Liu W (2016). GDF11 decreases bone mass by stimulating osteoclastogenesis and inhibiting osteoblast differentiation. Nat. Commun..

[26] Gao J (2020). Bone marrow mesenchymal stem cells improve bone erosion in collagen-induced arthritis by inhibiting osteoclasia-related factors and differentiating into chondrocytes. Stem Cell Res. Ther..

[27] Teramachi, J. et al. TAK1 is a pivotal therapeutic target for tumor progression and bone destruction in myeloma. *Haematologica* (2020). 10.3324/haematol.2019.234476 PMC8094086 32273474

[28] Nakashima K (2002). The novel zinc finger-containing transcription factor osterix is required for osteoblast differentiation and bone formation. Cell.

[29] Li H (2009). A novel microRNA targeting HDAC5 regulates osteoblast differentiation in mice and contributes to primary osteoporosis in humans. J. Clin. Investig..

[30] Park YS (2014). Selective osteogenesis by a synthetic mineral inducing peptide for the treatment of osteoporosis. Biomaterials.

[31] Yang Y (2020). Nedd4 ubiquitylates VDAC2/3 to suppress erastin-induced ferroptosis in melanoma. Nat. Commun..

[32] Reid IR (2020). A broader strategy for osteoporosis interventions. Nat. Rev. Endocrinol..

[33] Kim J (2011). Beraprost enhances the APC function of B cells by upregulating CD86 expression levels. J. Immunol..

[34] McKean JS (2015). The cAMP-producing agonist beraprost inhibits human vascular smooth muscle cell migration via exchange protein directly activated by cAMP. Cardiovasc. Res..

[35] Yu T (2020). Resveratrol promotes osteogenesis and alleviates osteoporosis by inhibiting p53. Aging.

[36] Jin F (2017). Bre enhances osteoblastic differentiation by promoting the Mdm2-mediated degradation of p53. Stem Cells.

[37] Wang ZW (2020). NEDD4 E3 ligase: functions and mechanism in human cancer. Semin. Cancer Biol..

[38] Ingham RJ, Gish G, Pawson T (2004). The Nedd4 family of E3 ubiquitin ligases: functional diversity within a common modular architecture. Oncogene.

[39] Zhang P (2015). G protein-coupled receptor 183 facilitates endothelial-to-hematopoietic transition via Notch1 inhibition. Cell Res..

[40] Fang NN (2014). Rsp5/Nedd4 is the main ubiquitin ligase that targets cytosolic misfolded proteins following heat stress. Nat. Cell Biol..

[41] Shao R (2016). Cdh1 regulates craniofacial development via APC-dependent ubiquitination and activation of Goosecoid. Cell Res..

[42] Kushioka J (2020). A novel negative regulatory mechanism of Smurf2 in BMP/Smad signaling in bone. Bone Res..

[43] Zou W (2011). The E3 ubiquitin ligase Wwp2 regulates craniofacial development through mono-ubiquitylation of Goosecoid. Nat. Cell Biol..

[44] Zhu W (2017). The E3 ubiquitin ligase WWP2 facilitates RUNX2 protein transactivation in a mono-ubiquitination manner during osteogenic differentiation. J. Biol. Chem..

[45] Kaneki H (2006). Tumor necrosis factor promotes Runx2 degradation through up-regulation of Smurf1 and Smurf2 in osteoblasts. J. Biol. Chem..

[46] Jiang Y, Wu W, Jiao G, Chen Y, Liu H (2019). LncRNA SNHG1 modulates p38 MAPK pathway through Nedd4 and thus inhibits osteogenic differentiation of bone marrow mesenchymal stem cells. Life Sci..

