# Supplementary figures and images for: Beraprost ameliorates postmenopausal osteoporosis by regulating Nedd4-induced Runx2 ubiquitination
Source: Cell Death Dis. 2021 May 15;12(5):497. doi: 10.1038/s41419-021-03784-8 (PMC8124066; doi:10.1038/s41419-021-03784-8)

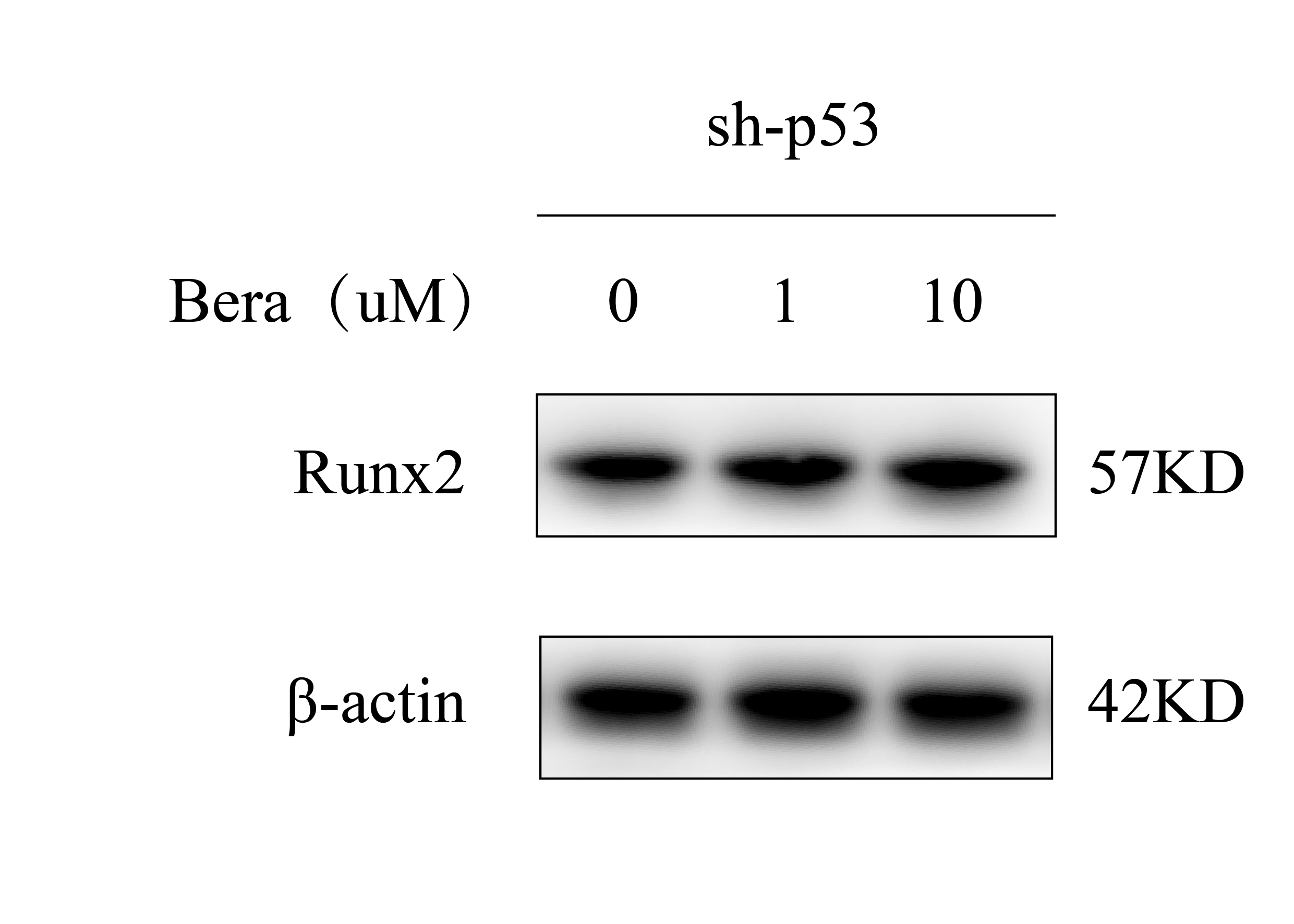

Supplement: Supplementary file 1 — supplemental figure [file 41419_2021_3784_MOESM1_ESM.tif]
