# Supplementary material for: Beraprost ameliorates postmenopausal osteoporosis by regulating Nedd4-induced Runx2 ubiquitination
Source: Cell Death Dis. 2021 May 15;12(5):497. doi: 10.1038/s41419-021-03784-8 (PMC8124066; doi:10.1038/s41419-021-03784-8)
Supplement: Supplementary file 2 — supplemental figure legend [file 41419_2021_3784_MOESM2_ESM.docx]

**Figure S Runx2 is almost unaffected by beraprost when p53 is knockdown** Western blotting images showing protein expression of Runx2 in beraprost-treated BMSCs when p53 is knockdown.
